# How to Elude the Doctor

**Published:** 1890-03

**Authors:** 


### HOW TO ELUDE THE DOCTOR.

A popular physician was recently called on by a friend, to whom, in the course of conversation, he said: "There are ten simple precautions which form an excellent rule of life, and if people would but observe them I should have to resort to some other means of making a livelihood." Then he enumerated the following: Don't read in street cars or other jolting vehicles. Don't pick the teeth with pins or other hard substances. Don't neglect any opportunity to insure a variety of food. Don't eat or drink hot and cold things immediately in succession. Don't pamper the appetite with such variety of food that may lead to excess. Don't read, write or do any delicate work unless receiving the light from the left side. Don't direct special mental or physical energies to more than eight hours' work in each day. Don't keep the parlor dark if you value your own and your children's health. Don't delude yourself into the belief that you are an exception so far as sleep is concerned; the nominal average of sleep is eight hours. Don't endeavor to rest the mind by absolute inactivity, let it rest in work in other channels, and thus rest the tired part of the brain.

UNCIVILIZED RUSSIA.—The most damaging facts presented by George Kennan in the *Century Magazine*, relative to the Russian penal system in Siberia, has been shockingly verified by recent outrages. The punishment of the official savage who ordered a delicate woman to be savagely flogged, should be of a piece with that inflicted upon his helpless victim. One more such outrage would totally extinguish the last spark of respect which civilized nations have for the Russian Autocracy. No subject is beneath the reach of justice and humanity; no official is too high to be made to answer for such a shameful abuse of his powers. The contempt and scorn of the civilized world are not enough for him. He should be made to smart.

